# Soft palate hematoma

**DOI:** 10.11604/pamj.2022.41.144.32021

**Published:** 2022-02-17

**Authors:** Krishna Prasanth Baalann, Nishanth Gurunathan

**Affiliations:** 1 Department of Community Medicine, Sree Balaji Medical College and Hospital, Bharath Institute of Higher Education and Research, Chennai, Tamil Nadu, India,; 2 Department of Oral Pathology and Microbiology, Sree Balaji Dental College and Hospital, Bharath Institute of Higher Education and Research, Chennai, Tamil Nadu, India

**Keywords:** Haemotoma, soft palate, swelling

## Image in medicine

Hematoma is an abnormal collection of blood outside the blood vessel. It occurs due to trauma or injury to the blood vessels. If there is great pressure within the blood vessel, the blood will continue to leak through the damaged wall and the hematoma will expand. Blood escaping from blood vessels is irritating to the surrounding tissue, may cause symptoms of inflammation including pain, swelling, and redness. A 36-year-old female presented with chief complaints of pain after eating a hot cooked fish instantly, which led to the formation of swelling. On intraoral examination, a painful hematoma and multiple lacerations in the soft palate were present. No active bleeding was present. The patient was hemodynamically stable. Painkillers were given for pain, the patient was advised for cold water gargling 3-4 times, after which the hematoma swelling reduced in size spontaneously. Betadine gargle (thrice daily) was advised, and the patient was requested to come for follow-up after one week.

**Figure 1:**
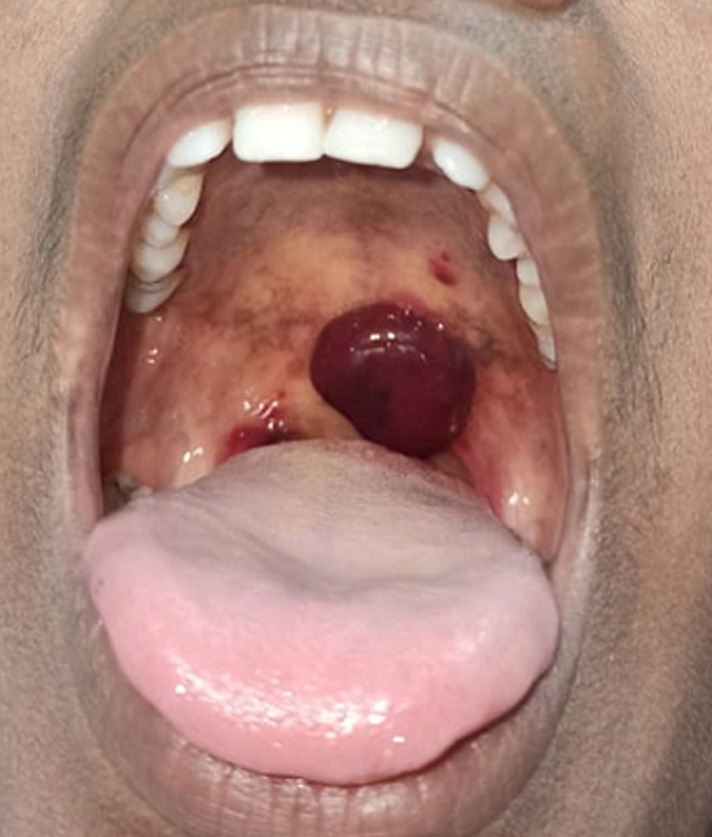
painful hematoma in soft palate

